# Mitochondrial quality control in intervertebral disc degeneration

**DOI:** 10.1038/s12276-021-00650-7

**Published:** 2021-07-16

**Authors:** Yu Song, Saideng Lu, Wen Geng, Xiaobo Feng, Rongjin Luo, Gaocai Li, Cao Yang

**Affiliations:** 1 Department of Orthopaedics, Union Hospital, Tongji Medical College, Huazhong University of Science and Technology, Wuhan, 430022 China; 2 Department of Ophthalmology, Union Hospital, Tongji Medical College, Huazhong University of Science and Technology, Wuhan, 430022 China

**Keywords:** Mitochondria, Autophagy

## Abstract

Intervertebral disc degeneration (IDD) is a common and early-onset pathogenesis in the human lifespan that can increase the risk of low back pain. More clarification of the molecular mechanisms associated with the onset and progression of IDD is likely to help establish novel preventive and therapeutic strategies. Recently, mitochondria have been increasingly recognized as participants in regulating glycolytic metabolism, which has historically been regarded as the main metabolic pathway in intervertebral discs due to their avascular properties. Indeed, mitochondrial structural and functional disruption has been observed in degenerated nucleus pulposus (NP) cells and intervertebral discs. Multilevel and well-orchestrated strategies, namely, mitochondrial quality control (MQC), are involved in the maintenance of mitochondrial integrity, mitochondrial proteostasis, the mitochondrial antioxidant system, mitochondrial dynamics, mitophagy, and mitochondrial biogenesis. Here, we address the key evidence and current knowledge of the role of mitochondrial function in the IDD process and consider how MQC strategies contribute to the protective and detrimental properties of mitochondria in NP cell function. The relevant potential therapeutic treatments targeting MQC for IDD intervention are also summarized. Further clarification of the functional and synergistic mechanisms among MQC mechanisms may provide useful clues for use in developing novel IDD treatments.

## Lower back pain: Disk degeneration and mitochondrial quality

Dysfunctional mitochondria, the powerhouses of cells, are associated with development of intervertebral disc degeneration (IDD) and researchers are studying the mechanisms of mitochondrial quality control (MQC) to identify better treatments. IDD is a common and early-onset condition and one of the causes of low back pain. Current treatments address symptoms, not root causes, and a better understanding of IDD is needed. Cao Yang at Huazhong University of Science and Technology, Wuhan, China, and coworkers reviewed how cells maintain healthy mitochondria and the role of mitochondria in the development of IDD. The mechanisms of MQC include removing damaged mitochondria, generating new mitochondria, and managing harmful mitochondrial products. Treatments that target MQC show therapeutic promise, and further investigation of the roles played by the different mechanisms of MQC could lead to better treatments for this debilitating condition.

## Introduction

Low back pain (LBP) is an extremely prevalent musculoskeletal disorder worldwide, and almost everyone suffers an acute or chronic episode of LBP during their lifetime^1^. According to the statistical analysis of the Global Burden of Disease Study 2017, LBP was consistently the leading contributor to the increase in years lived with a disability^2^. Intervertebral disc degeneration (IDD), which results in progressive spinal deformity, stenosis, and the inflammatory response, has been identified as a well-known contributor to LBP^3,4^. Nevertheless, current clinical therapeutic strategies, known as conservative treatment and surgical intervention, aimed at alleviating symptoms rather than targeting IDD directly^1^. Further clarifying the molecular mechanism of IDD will provide a new approach for precise intervention strategies for LBP.

Normal intervertebral discs consist of gelatinous nucleus pulposus (NP) as the central structure, surrounded by lamellar annulus fibrosus (AF) and sandwiched by superior and inferior cartilaginous endplates (CEPs)^5^. The gelatinous NP tissue is critical for the physiological function of intervertebral discs to absorb and disperse mechanical loadings during spinal motion, such as flexion, extension, bending, and rotation^5^. Currently, NP cells are identified as the main cell group that undergoes anabolic and catabolic metabolism and maintains extracellular matrix homeostasis. Mounting evidence has demonstrated that the loss of function of NP cells through senescence, apoptosis, necroptosis, inflammatory response, or phenotypic change plays important roles in inducing the dehydration of NP tissue and promoting IDD progression^6–8^.

**Fig. 1:**
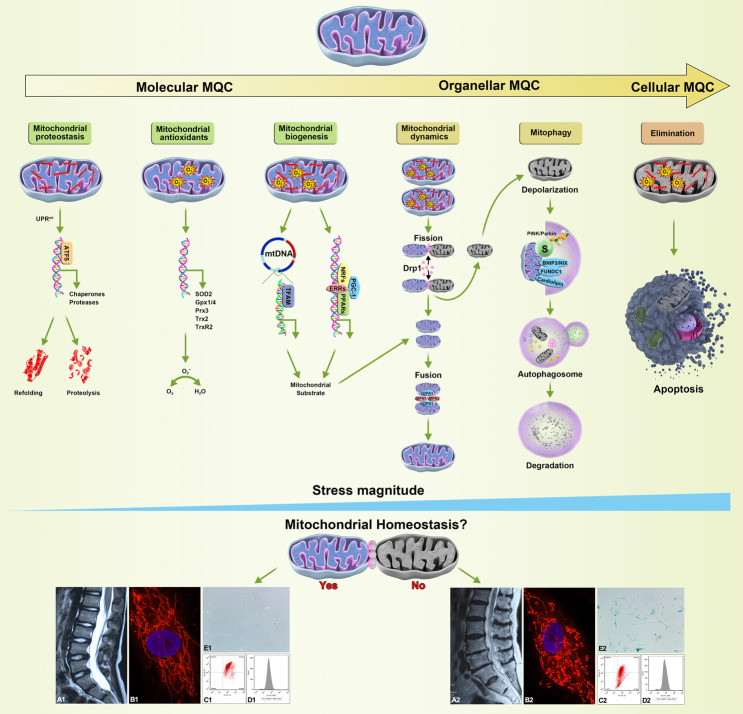
The molecular, organellar, and cellular levels of MQC strategies that maintain mitochondrial homeostasis. With increasing stress magnitude, multilevel and well-orchestrated MQC strategies are implemented. Mitochondrial proteostasis is monitored by UPR^mt^ activity and executed by ATF5, which mainly promotes the expression of mitochondrial chaperones and proteases that conduct the refolding or proteolysis of misfolded and damaged proteins. Under primary and secondary oxidative stress, mitochondrial antioxidant members (SOD2, Gpx1/4, Prx3, Trx2, TrxR2) eliminate superoxide radicals and maintain redox homeostasis. Further damage can induce the selective separation of healthy and injured mitochondria by Drp1-dependent fission. The remaining intact daughter mitochondria are replenished by mitochondrial biogenesis involving mitochondrial transcription factor-mediated (TFAM) mtDNA and nuclear transcription factor-mediated (PGC-1, NRFs, ERRs, PPARs) DNA expression and integrated by OPA1/MFN-dependent fusion, while the disrupted daughter mitochondria are swallowed and degraded by mitophagy, which depends on ubiquitinated mitochondrial substrates (S) in the PINK1/Parkin ubiquitin pathway or other mitophagy receptors (BNIP3/NIX, FUNDC1, or cardiolipin). Finally, irreversible damage to mitochondria induces devastating effects on cellular bioactivities and results in apoptosis. Healthy MQC strategies succeeded in maintaining good intervertebral disc morphology (A1) and mitochondrial homeostasis through mitochondrial elongation and integral structure (B1), high mitochondrial membrane potential (C1), low ROS levels (D1), and fine-tuned NP cell status (E1). Defective MQC strategies aggravate intervertebral disc morphologic disruption (A2) and fail to maintain mitochondrial homeostasis and exhibit mitochondrial fragmentation (B2), low mitochondrial membrane potential (C2), high ROS levels (D2), and poor NP cell status (E2). Interpretively, intervertebral disc morphology was defined on the basis of T2-weighted magnetic resonance imaging (A1/A2), mitochondrial structure as assessed with MitoTracker Red CMXRos staining (B1/B2), mitochondrial membrane potential as determined by JC-1 assay (C1/C2), ROS levels as measured using 2′,7′-dichlorofluorescin diacetate staining (D1/D2), and NP cell status as assessed using senescence-associated β-galactosidase staining (E1/E2).

Although the intervertebral disc has been identified as the largest avascular organ and found to generate energy through anaerobic glycolysis^9,10^, a critical role for mitochondria in promoting metabolic adaptation has been suggested^11,12^. In addition to material and energy metabolism, mitochondria also participate in regulating the function of second messengers, such as reactive oxygen species (ROS) and calcium, and the activation of various signaling pathways, which play important roles in regulating cellular function and determining cell fates^13^. In IDD progression, structural and functional abnormalities in mitochondria have been observed in NP cells^6,14,15^. Mitochondrial quality control (MQC), involving molecular, organellar, and cellular level mechanisms, is considered a critical surveillance and protective system for limiting mitochondrial damage and ensuring mitochondrial integrity^16^ (Fig. 1). Dysfunctional MQC strategies and aggravated mitochondrial damage are considered major contributing factors in promoting NP cell function loss^6,17^.

In this review, we summarize defective MQC strategies linked to mitochondrial dysfunction that contribute greatly to NP cell function loss and IDD progression. Relevant molecular strategies with clinical translational potential that might be developed for the prevention and treatment of IDD progression are also discussed.

## Mitochondrial dysfunction and IDD pathogenesis

Although in some studies, few mitochondria were detected in adult NP tissue specimens^14,18^, well-developed mitochondria exist in NP cells, especially in fetal NP cells^19–21^. Multiple studies have confirmed that the etiological factors in IDD pathogenesis are involved in endogenous genetic predisposition and exogenous stressors, such as decreased nutrient transport, mechanical overloading, diabetes, smoking, infection, and aging^4^. Interestingly, all these etiological mechanisms are associated with mitochondrial damage, which has been proposed to underlie the pathophysiology of NP cell function loss and IDD progression.

First, the early degenerative process of notochord-like NP cells due to phenotypic changes or cell death was partially attributed to loading, avascularity, and the hypoxic and hyperglycemic microenvironment, in which increased mitochondrial fission, fragmentation, mitophagy and mitochondrial damage play adaptive and aggravating roles^22–26^. Due to the origination of the notochord, NP cells present notochordal markers, also called notochord-like NP cells, and are regarded as indicative of the initial cell phenotype in NP tissue^27–30^. With disc maturation, the supplies of nutrients and oxygen were diminished, especially in the central NP tissue, which induced the loss of the notochord-like phenotype in NP cells, partially due to its more active metabolism and greater susceptibility to nutrient deprivation, compared with chondrocyte-like NP cells^24^. Mitochondria loss may be an adaptive process. Similarly, the absence of a master regulator adapting the cells to hypoxia, namely, hypoxia-inducible factor (HIF)-1α, may also promote cell death and NP dysplasia, which is accompanied by disordered mitochondrial oxidative metabolism and mitophagy^31–33^. Loading stress can also promote the transition of notochord-like NP cells to chondrocyte-like NP cells or induce the apoptotic death of notochord-like NP cells, in which a mitochondrial pathway plays a partial role^23,26,34^. In diabetes-associated diseases, hyperglycemia can disrupt mitochondrial function and induce notochordal cell death^25,35^. Recently, targeting notochordal cells as regenerative strategies of NP tissue has been recommended, and this strategy includes notochordal cell coculture, notochordal cell-conditioned medium, and notochordal cell matrix^36–41^. Whether the function of notochord-like NP cells can be rescued by modeling mitochondrial function needs to be determined through more investigation.

Second, NP cell function loss occurs in the IDD progression stage due to cell senescence, death, inflammatory responses, and imbalances in anabolic and catabolic metabolism, which are also closely associated with mitochondrial damage^8,42–49^. In addition to the deficiency of energy generation, damaged mitochondria negatively affect cellular function by releasing various deleterious molecules. Studies have confirmed that many risk factors associated with IDD progression, including mechanical overloading, inflammation, nutrition deprivation, and the accumulation of advanced glycation end products (AGEs) or progerin, can cause excessive ROS production, calcium disorders, and the leakage of pro-death factors such as cytochrome C and apoptosis-inducing factor^6,8,17,47,48,50,51^. These hazardous molecules can act alone or jointly, composing a complex network in the mechanism of IDD pathogenesis. Although various ROS scavengers, antioxidants and calcium chelators can inhibit NP cell senescence or death in experiments conducted in vitro and alleviate IDD progression in experimental animal models^52–55^, none of these agents have been translated into clinical application with satisfactory therapeutic efficiency in patients with IDD or IDD-related complications. These studies implied that the release of harmful molecules was merely a consequence of mitochondrial disruption, and targeting these damaging molecules may require salvage therapies, not fundamental strategies. In contrast, a pool of healthy mitochondria maintained by an elaborate MQC system seems to lay a better foundation for controlling the production of mitochondrial-derived danger molecules. Presumably, this control can be achieved by elucidating and manipulating the critical MQC strategies in specific pathogeneses to achieve efficient coordination of mitochondrial metabolism, incidental mitochondrial damage, and mitochondrial regeneration. Some studies have attempted to investigate precise MQC strategies and elucidate the corresponding therapeutic targets (Table 1).

**Table 1:** Defective MQC strategies are attributed to IDD progression induced by various risk factors.

Study (year)	Stressors/Interventions	Models	Key findings	Refs
*Mitochondrial proteostasis*				
Chooi et al.^65^	Mechanical loading	NP cells in 3D collagen	Longer loading duration resulted in a continuous upregulation of the Hsp70 gene, which might play a role in cell survival following mechanical stress.	^65^
Chooi et al.^66^	Mechanical loading	Disc organ culture	Static loading induced higher Hsp70 expression than dynamic loading in NP cells.	^66^
Gogate et al.^67^	Low oxygen	NP cell culture	Hsp70, HIFs, and TonEBP form a regulatory loop to adapt the NP cells to the unique hypoxic and hyperosmolar microenvironment.	^67^
Tasi et al.^68^	Hypertonic milieu	NP cell culture	Hypertonicity enhanced the expression of Hsp70 via the activation of the ERK and p38-MAPK pathways.	^68^
*Mitochondrial antioxidants*				
Chen et al.^77^	TNF-α/Rg3 treatment	NP cell culture	TNF-α treatment led to the reduction in SOD and GSH-PX activity levels, which can be reversed by bioactive extract Rg3.	^77^
Jin et al.^78^	Estrogen depletion	Rat disc	Ovariectomized rats showed decreased activities of SOD and GSH-Px and an increased **GSSG/GSH** ratio.	^78^
Song et al.^6^	AGEs	Rat disc and NP cell culture	AGE treatment decreased the protein levels of SOD2, TRX2, **T**RXR2 and catalase in NP cells.	^6^
Chen et al.^49^	Mechanical loading	NP cell culture	Mechanical loading induced a downward trend in SOD activity.	^49^
Jiao et al.^79^	Hypertonic milieu	NP cell culture	Hyperosmolarity culture significantly decreased the total SOD activity compared with the in situ-osmolarity culture.	^79^
Gu et al.^80^	Lipopolysaccharides	NP cell culture	NP cells treated with LPS exhibited reduced SOD and catalase activity levels.	^80^
Tang et al.^81^	H2O2/Honokiol	NP cell culture	Honokiol pretreatment significantly reversed the H2O2-suppressed expression of the SOD and Gpx1 genes.	^81^
He et al.^82^	H2O2/Melatonin	NP cell culture	Melatonin pretreatment restored higher levels of GSH and SOD activity under H2O2 conditions.	^82^
Chu et al.^83^	H2O2/Plumbagin	NP cell culture	Plumbagin significantly increased the GSH content, as well as the activity of catalase, SOD and GSH-Px.	^83^
Dong et al.^84^	Lipopolysaccharides/Pilose antler peptide	NP cell culture	Pilose antler peptide attenuated the decrease in SOD induced by lipopolysaccharides challenge in a concentration-dependent manner.	^84^
*Mitochondrial dynamics*				
Xu et al.^17^	Sulforaphane/Progerin	Mice disc and NP cell culture	NP cells showed the increased expression level of Drp1 and decreased levels of Mfn1/2 in the progerin group, indicating less fusion and more fission in mitochondria.	^17^
Kang et al.^52^	Mechanical loading/MitoQ	Disc organ and NP cell culture	Mechanical loading promoted the mitochondrial translocation of Drp1 and upregulated Drp1, Mff and Fis1 protein levels, whereas MitoQ could alleviate this process.	^52^
Xu et al.^50^	IL-1β/NaHS	NP cell culture	NaHS pretreatment significantly decreased the mitochondrial translocation of Drp1 induced by IL-1β in NP cells.	^50^
*Mitophagy*				
Kang et al.^52^	Mechanical loading/MitoQ	Disc organ and NP cell culture	MitoQ promotes PINK1/Parkin‐mediated mitophagy and repairs defective mitophagic flux in human NP cells exposed to mechanical loading.	^52^
Zhang et al.^104^	TNF-α/Salidroside	NP cell culture	Parkin was upregulated in degenerative NP tissues in vivo as well as in TNF-α stimulated NP cells in vitro, and salidroside could enhance Parkin expression and eliminate mitochondria damage.	^104^
Chen et al.^105^	TBHP	Rat disc and NP cell culture	Mfn2 overexpression stimulates an ROS-dependent mitophagy via PINK1/Parkin pathway in TBHP-treated rat NP cells.	^105^
Chen et al.^106^	TBHP/Melatonin	NP cell culture	TBHP suppressed mitophagy activity, while melatonin effectively reversed TBHP‐induced NP cell apoptosis via Parkin‐dependent mitophagy induction.	^106^
Xie et al.^107^	TBHP/circERCC2	Rat disc and NP cell culture	circERCC2 can ameliorate IVDD through miR-182-5p/SIRT1 axis by activating the mitophagy pathway (PINK1, Parkin, P62, and LC3II/I).	^107^
Wang et al.^108^	TBHP/Honokiol	NP cell culture	Honokiol can facilitate the colocalization of LC3 and Bnip3L and enhance mitophagy in TBHP-treated NP cells by enhancing SIRT3.	^108^
Xu et al.^32^	TBHP	NP cell culture	TBHP induced parkin-dependent excessive mitophagy that was detrimental for NP cell survival.	^32^
*Mitochondrial biogenesis*				
Hua et al.^117^	H2O2/Icariin	NP cell culture	Icariin activates the NRF1/2 and TFAM signaling pathways and mitochondrial biogenesis.	^117^
Song et al.^6^	AGEs	Rat disc and NP cell culture	AGE treatment decreased the activation of the AMPK/PGC-1α pathway in NP cells.	^6^
Kang et al.^52^	Mechanical loading/MitoQ	Disc organ and NP cell culture	MitoQ inhibited Keap1 expression, which activated the Nrf2 signaling cascade in human NP cells.	^52^
Wang et al.^108^	TBHP/Honokiol	NP cell culture	Honokiol can effectively upregulate SIRT3 expression via the AMPK-PGC-1α signaling pathway in NP cells.	^108^

### The MQC system in IDD pathogenesis

#### Mitochondrial proteostasis

Generally, the unfolded protein response (UPR) is referred to as a protective signaling pathway that dissolves accumulated damaged and/or unfolded/misfolded proteins and reestablishes cellular proteostasis^56,57^. In mitochondria, a diverse number of stressors that impair mitochondrial function can induce the mitochondrial unfolded protein response (UPR^mt^), mediating adaptive transcriptional activity and promoting the recovery of the mitochondrial network^58,59^. First, the increased expression of mitochondria-localized molecular chaperones induced by UPR^mt^ activity, such as Hsp70 and Hsp60, not only can facilitate the correct folding of newly synthesized proteins but can also unfold and disaggregate misfolded proteins^60,61^. Second, the UPR^mt^ can also enhance the expression of a significant number of mitochondrial proteases, such as LONP1 and CLPP, which are important for protein maintenance and elimination of oxidized and damaged proteins^62^. In addition, the UPR^mt^ can promote the expression of nuclear-encoded detoxification enzymes and mitochondrial protein-imported components^63,64^.

In one model of collagen microencapsulation, the stress response of NP cells to compression loading was examined^65^. It was found that longer loading durations significantly upregulated the Hsp70 level and had little influence on the apoptosis ratio of the NP cells, which indicated a protective effect following mechanical stress^65^. In addition, this research team also used adult bovine caudal discs to construct an organ culture model and found that Hsp70 expression appeared to be upregulated immediately after loading and was decreased upon resting following a repeated loading cycle in NP cells but not in AF cells^66^. In addition, a hypoxic or hypertonic microenvironment showed dramatic effects on mitochondrial function in NP cells^21,33^^,53^, in which the levels and function of the mitochondrial chaperone Hsp70 presented a corresponding response^67^^,68^. Shilpa et al.^67^ found that tonicity enhancer-binding protein (TonEBP) and HIFs are involved in some interaction that leads to the regulation of Hsp70 levels, while Hsp70 function also negatively regulates HIF-1α protein stability and transcriptional activity. Studying the hyperosmotic microenvironment, Tsai et al^68^ confirmed an adaptive response of NP cells to hyperosmotic stress depending on the activity of the important osmoregulator TonEBP, and its target gene, Hsp70, contributed greatly to this response. Thus, the mitochondrial chaperone Hsp70 plays critical role in regulating mitochondrial function and adaptive responses of NP cells to various stresses. The complex function of UPR^mt^ involves many upstream regulators and downstream mediators. Future studies need to fully characterize the UPR^mt^ process in the progression of IDD pathogenesis and NP cell function loss.

#### Mitochondrial antioxidant system

Along with mitochondrial respiration and metabolism, ROS are commonly generated, such as superoxide anion and hydrogen peroxide (H_2_O_2_)^69,70^. The antioxidant system in mitochondria largely contributes to monitoring and controlling cellular ROS levels. Multiple antioxidant enzymes participate in the construction of the mitochondrial antioxidant system, including superoxide dismutases (SODs), glutathione peroxidases (Gpxs), peroxiredoxins (Prxs), and some enzymes that exhibit redundant actions^70–72^. SODs can dismutate superoxide anions to H_2_O_2_, and SOD2 is mainly located in mitochondria^71^. Subsequently, Gpxs/Prxs can catalyze H_2_O_2_ to H_2_O, mainly through Gpx1/4, Prx3, Trx2, and TrxR2, in mitochondria^72^. A healthy antioxidant system is crucial in determining whether ROS are playing “friends” or “foes” roles, which partially depends on their concentration, location, and functional context^73,74^. Appropriate or low ROS levels contribute to multiple essential biochemical signaling processes ranging from cell metabolism to microorganism defense. Excessive generation and/or inadequate elimination of ROS results in oxidative damage to molecules and oxidative stress, which has been implicated in various pathogeneses and, in the case of cancer, in roles that protect tumors from elimination.

In IDD pathogenesis, it has been demonstrated that various risk factors, such as mechanical overloading, AGE accumulation, nutrient deprivation, and inflammatory cytokines, can promote IDD events by inducing oxidative stress^6,42,52,75,76^. Under compression conditions, NP cells showed significantly decreased protein levels of SOD2, which could be rescued by MitoQ treatment, and they showed antioxidant function^52^. It was found that the reduction in SOD and Gpx activity levels was involved in TNF-α-induced oxidative stress in human NP cells and that the rescue of SOD and Gpx activity levels by administration of ginsenoside Rg3 reversed this degenerative process^77^. In ovariectomized animal models, IDD progression was associated with disrupted redox homeostasis involving the functional loss of SOD, Gpx, and GSSG/GSH balance^78^. Estrogen supplementation can enhance antioxidant capacity and correct redox imbalance stress^78^. In addition, the partial or whole functional deficiency of mitochondrial antioxidant enzymes is also critical in many other IDD processes induced by hyperosmolarity, mechanical overloading, AGE accumulation, or lipopolysaccharide^6,49,79,80^. The effects of biologically active components used to treat IDD have greatly improved in recent years. It has been confirmed that honokiol, nicotinamide mononucleotide, melatonin, plumbagin, and pilose antler peptide can efficiently enhance the catalytic activity of SOD and/or Gpx, which contributes substantially to resistance to oxidative stress and IDD progression^6,81–84^. More investigation and clarification targeting the mitochondrial antioxidant system will provide clearer strategies for IDD intervention.

#### Mitochondrial dynamics

Dynamic mitochondria are identified by high fusion and fission activities, ensuring their efficient response to changing requirements for energy production, calcium homeostasis, lipid biogenesis, fatty acid synthesis, and responses to stress conditions^85^. In quiescent cells, increased mitochondrial fission converts functionally mature mitochondrial networks into immature states that are applicable for conditions of low metabolic demand and reduced oxidate exposure^86^. Under stress or proliferative conditions, however, activated mitochondrial fusion activity can maximize the oxidative capacity for energy demand and increase the degree of cross-complementation for attenuating defective mitochondria, enhancing their response capacity^87–89^. A healthy balance of dynamic mitochondrial activity is well regulated by large dynamin-related guanosine triphosphatases (GTPases)^90^. Long-form optic atrophy1 (L-Opa1) and mitofusin1/2 (Mfn1/2) are correspondingly critical for inner and outer mitochondrial membrane fusion, and dynamin-like protein (Drp1) and short-form Opa1 (S-Opa1) are correspondingly critical for outer and inner mitochondrial membrane fission. In addition, other factors have also been found to assemble the fusion and fission machinery, such as mitoPLD, FIS1, MFF, MiD49, and MTP18^90^. Both innate mutations and acquired stressors that disturb the healthy fusion and fission machinery can induce pathological mitochondrial dynamics^91–93^.

It has been proven that disrupted mitochondrial dynamics are also closely related to mitochondrial dysfunction and oxidative stress in the IDD process. Xu et al found that progerin accumulation in human NP tissues was associated with IDD progression, and further study confirmed that progerin stimulation can shift mitochondrial dynamics toward fission events by decreasing the levels of mitochondrial fusion factors Opa1 and Mfn1/2 and increasing the levels of the mitochondrial fission factor Drp1^17^. By rescuing the balance of mitochondrial dynamics, sulforaphane can significantly attenuate progerin-induced mitochondrial dysfunction and NP cell senescence^17^. Using a mechanical overloading model of IDD, Kang et al observed that the migration of Drp1 from the cytoplasm to mitochondria was significantly enhanced. Similarly, the total protein levels of Drp1, Mff, and Fis1 were significantly upregulated and those of Opa1 and Mfn1/2 were downregulated by mechanical overloading, which largely contributed to NP cell damage and was ameliorated by MitoQ intervention^52^. Similarly, hydrogen sulfide treatment can also decrease the mitochondrial membrane location of Drp1 and mitochondrial dysfunction induced by proinflammatory factors^50^. These results may suggest that rebalancing healthy mitochondrial fusion/fission dynamics is critical for IDD intervention.

#### Mitophagy

In contrast to bulk autophagy, in which cellular components are recycled to meet nutrient demand, selective autophagy functions to clear unwanted and damaged substances^94^. Autophagy that is specific to mitochondria is referred to as mitophagy, which is crucial for the elimination of damaged or superfluous mitochondria^95,96^. The principal mechanisms of selective autophagy rely on the use of specialized cargo-binding adaptor proteins, also called selective autophagy receptors^97^. Members of the PARK family, PARK2 and PARK6, which encode the E3-Ub ligase PARKIN and the mitochondrial-targeted kinase PINK1, respectively, were shown to be key mediators of mitophagy and mitochondrial surveillance. The accumulation of PINK1 on the mitochondrial surface is the first step to sensing mitochondrial stress. Depolarization of the mitochondrial membrane, decreasing its potential, prevents the import of PINK1 through the mitochondrial membrane and secondary proteolytic cleavage, which leads to the effective accumulation of full-length PINK1 and its activating autophosphorylation. Activated PINK1 can phosphorylate ubiquitin and elicit PARKIN recruitment to the mitochondrial surface. Ultimately, the PINK1-PARKIN pathway results in ubiquitinated mitochondrial substrates (S), autophagy receptor recruitment, and clearance of damaged mitochondria^98–100^. In addition to the PINK1-PARKIN pathway, several selective mitochondrial autophagy receptors have been identified: BNIP3/NIX, FUNDC1, NLRX1, and PHB2^100,101^. Some lipid autophagy receptors have also been identified^102,103^. Cardiolipin, a lipid unique to mitochondria, has recently been reported to mediate mitophagy^103^.

It has been observed that the mitophagy process in NP cells can be induced by mechanical loading, TNF-α expression, and exogenous hydroperoxide treatment, which is beneficial for alleviating NP cell senescence, preventing cell death, and correcting imbalances in anabolic/catabolic metabolism^52,104,105^. Based on this, targeting the PINK1-PARKIN pathway by natural extracts, such as salidroside and melatonin, may promote effective mitophagy and its beneficial effects^104,106^. Noncoding RNAs targeting molecules upstream of the PINK1-PARKIN pathway also have been shown to exert a positive effect on mitophagy^107^. In addition, other mitophagy receptors, such as BNIP3, were found to mediate the selection and elimination of damaged mitochondria and promote the resistance of NP cells to oxidative stress^108^. The outcome of mitophagy as beneficial or detrimental in disease progression and/or intervention is a cellular factor- and context-dependent^109,110^. Under strong oxidative conditions, excessive mitophagy is induced in NP cells, which contributes greatly to the promotion of apoptotic cell death^32^. This study also identified the critical role of HIF-1α/NDUFA4 L2 in repressing excessive mitophagy and alleviating NP cell apoptosis^32^. Thus, more clarification of mitophagy mechanisms in IDD progression may provide precise therapeutic targets for driving the beneficial effect of mitophagy in IDD intervention.

#### Mitochondrial biogenesis

Mitochondrial biogenesis is an important process that promotes the synthesis of new mitochondria through expansion and division of pre‐existing mitochondria, which reach and maintain mitochondrial homeostasis by obtaining a balance between mitochondrial fission and fusion and realizing mitochondrial turnover through mitophagy^111^. As semiautonomous organelles, mitochondrial proteins not only transcribe and translate nuclear and mitochondrial genomes but also synchronize and coordinate their expression^112^. Multiple transcription factors and coactivators have been shown to orchestrate genome expression during mitochondrial biogenesis^113^. Nuclear respiratory factors (NRF1/2) were the first nuclear transcription factors found to be involved in the transcription of several mitochondrial genes that mainly encode subunits of mitochondrial respiratory chain complexes^114^. Members of the nuclear receptor (NR) superfamily, mainly peroxisome proliferator-activated receptors (PPARs) and estrogen-related receptors (ERRs), are also important for controlling the transcription of nuclear genes encoding mitochondrial substrates involved in FAO, the TCA cycle, and ETC/OXPHOS^115^. In addition, peroxisome proliferator-activated receptor-gamma coactivator-1 (PGC-1) coactivators build regulatory circuitry and serve as central components to control the transcriptional activities of NRF and NR family proteins^115^. PGC-1/NRF-1 coactivation is also critical for the expression of TFAM, a transcription factor of the mitochondrial genome encoding 13 components of OXPHOS system complexes^116^. Hua et al.^117^ observed that icariin can significantly rescue mitochondrial function in human NP cells by activating the NRF1/2 and TFAM pathways and promoting mitochondrial biogenesis. Other studies have also observed the functions of PGC-1 and NRFs in protecting mitochondria against damage in NP cells^6,52,108^, but whether mitochondrial biogenesis is involved in this protection remains to be elucidated.

#### Cellular elimination of mitochondria

In the case of sustained mitochondrial stress and irreversible mitochondrial damage, the cellular quality control mechanisms that rely on apoptosis and the turnover of entire cells are engaged to ensure the homeostasis of the organism. Otherwise, damaged mitochondria would be transferred to neighboring cells, and malfunctioning cells can accumulate and lead to tumor development and other pathological conditions^118,119^. Indeed, extensive mitochondrial damage promotes prolonged mitochondrial outer membrane permeabilization (MOMP), which can contribute to the release of proapoptotic molecules such as cytochrome c, Diablo, HtrA2, and AIF from the mitochondrial intermembrane space into the cytosol, resulting in caspase activation and apoptosis^120^. The well-characterized mechanism that invokes MOMP is dependent on Bax-Bak oligomerization and translocation to the mitochondrial outer membrane where pores or channels are formed to generate MOMP. In addition, this process is also affected by pro-survival members of the Bcl-2 family (Bcl-2, Bcl-xL), which either bind to Bax-Bak and prevent their oligomerization or are “neutralized” by the restraint of BH3-only proteins (Bid and Bad); the outcome of Bcl-2 family proteins dictates whether a cell survives or undergoes apoptosis^121^. Another model of channel formation is mediated by voltage-dependent anion channel (VDAC), the most abundant protein of the mitochondrial outer membrane^109^. Furthermore, crosstalk between VDAC and members of the Bcl-2 (homology) family have also detected and found to function together to mediate cytochrome c release and cell apoptosis. Thus, damaged mitochondrial substrates promote cell apoptosis that eliminates whole cell contents, including damaged mitochondria, at the cellular level of MQC. In the progression of IDD, mitochondria-derived signals have been confirmed to promote Bax-Bak oligomerization and further induce MOMP and NP cell apoptosis in various risk factor-induced degenerative models^6,122^. However, recent studies have mostly focused on the effect of damaged mitochondria on NP cell apoptosis, but not the other forms of cell death. Recently, multiple studies demonstrated various cell death models in the IDD process, including ferroptosis^123^, pyroptosis^124^, and necroptosis^125^, all of which are closely associated with mitochondrial damage. Cells commit suicide at the appropriate time as part of the natural cell turnover process that is essential to optimal tissue functioning, while more investigation and clarification between mitochondria-associated cell death and the cellular level of MQC are needed.

## Concluding remarks and future perspectives

**Fig. 2:**
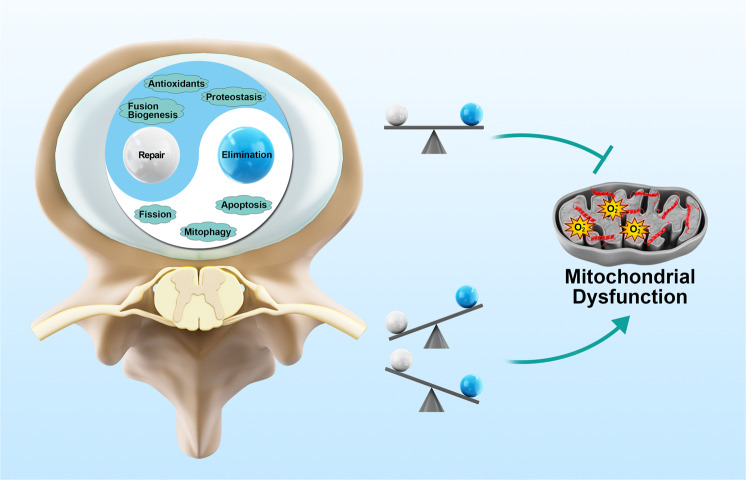
The well-orchestrated coordination and balance among MQC strategies are essential for protecting against mitochondrial dysfunction. Mitochondrial proteostasis, antioxidants, biogenesis, and fusion work to maintain the healthy status of existing mitochondria or to generate new mitochondria. In parallel, mitochondrial fission, mitophagy, and apoptotic elimination lead to the separation and removal of old and damaged mitochondria. Either incompatible repair or elimination activities can promote mitochondrial dysfunction.

MQC is tightly associated with adaptive changes in mitochondrial metabolism and the timely elimination of mitochondrial damage. Multiple levels of MQC action and surveillance strategies were involved in the mitochondrial adaptive response and defective mitochondrial elimination for the maintenance of mitochondrial homeostasis. Indeed, well-orchestrated coordination and balance within MQC functional strategies efficiently maintain healthy mitochondrial metabolism (Fig. 2). For instance, eliminating damaged mitochondrial proteins by proteases or mitophagy and renewing components by adding protein and lipids through biogenesis or UPR^mt^ enables the monitoring and regulation of mitochondrial protein quality^112^. The mitochondrial network can be fragmented and segregated by fission, which requires synergistic fusion of healthy fragments and selective mitophagy to eliminate damaged mitochondrial fragments^126^. In addition, mitophagy must be coordinated with new mitochondrial production through fusion/fission and biogenesis to control mitochondrial mass^127,128^.

Abnormal MQC function was consistently observed in the IDD process. Each mechanism of MQC, including UPR^mt^, mitochondrial antioxidants, mitochondrial dynamics, mitophagy, and biogenesis, functions in regulating NP cell death, senescence, the inflammatory response, and anabolic and catabolic metabolism. Targeting one or several mechanisms of MQC has shown therapeutic potential for IDD intervention. However, the relative contribution of each mechanism to IDD progression remains largely unknown. In addition, crosstalk between different mechanisms also exists, and it is essential to identify the molecular underpinnings that regulate and coordinate these processes to achieve optimal MQC, thereby reducing NP cell function loss and IDD progression. Further investigation aiming to understand the molecular mechanisms and develop therapeutic strategies individually or in combination that target efficient MQC processes is expected.

## Data Availability

The authors confirm that all data are fully available without restriction. All relevant data are described within the paper.

## References

[1] Deyo RA, Mirza SK (2016). CLINICAL PRACTICE. Herniated Lumbar Intervertebral Disk. The. N. Engl. J. Med..

[2] Collaborators GDaIIaP. (2018). Global, regional, and national incidence, prevalence, and years lived with disability for 354 diseases and injuries for 195 countries and territories, 1990-2017: a systematic analysis for the Global Burden of Disease Study 2017. Lancet.

[3] Vlaeyen JWS (2018). Low back pain. Nat. Rev. Dis. Prim..

[4] Risbud MV, Shapiro IM (2014). Role of cytokines in intervertebral disc degeneration: pain and disc content. Nat. Rev. Rheumatol..

[5] Roughley PJ (2004). Biology of intervertebral disc aging and degeneration: involvement of the extracellular matrix. Spine.

[6] Song Y (2018). Sirtuin 3-dependent mitochondrial redox homeostasis protects against AGEs-induced intervertebral disc degeneration. Redox Biol..

[7] Kang L (2019). Restoration of autophagic flux rescues oxidative damage and mitochondrial dysfunction to protect against intervertebral disc degeneration. Oxid. Med. Cell. Longev..

[8] Song Y (2017). Advanced glycation end products regulate anabolic and catabolic activities via NLRP3-inflammasome activation in human nucleus pulposus cells. J. Cell. Mol. Med..

[9] Grunhagen T, Wilde G, Soukane DM, Shirazi-Adl SA, Urban JP (2006). Nutrient supply and intervertebral disc metabolism. J. Bone Jt. Surg. Am..

[10] Urban JP, Smith S, Fairbank JC (2004). Nutrition of the intervertebral disc. Spine.

[11] Martin JA (2012). Mitochondrial electron transport and glycolysis are coupled in articular cartilage. Osteoarthr. Cartil..

[12] McElroy GS, Chandel NS (2017). Mitochondria control acute and chronic responses to hypoxia. Exp. cell Res..

[13] Görlach A, Bertram K, Hudecova S, Krizanova O (2015). Calcium and ROS: a mutual interplay. Redox Biol..

[14] Hartman R (2018). Age-dependent changes in intervertebral disc cell mitochondria and bioenergetics. Eur. Cells Mater..

[15] Fernando HN (2011). Mechanical loading affects the energy metabolism of intervertebral disc cells. J. Orthop. Res..

[16] Andreux PA, Houtkooper RH, Auwerx J (2013). Pharmacological approaches to restore mitochondrial function. Nat. Rev. Drug Discov..

[17] Xu X (2019). Progerin accumulation in nucleus pulposus cells impairs mitochondrial function and induces intervertebral disc degeneration and therapeutic effects of sulforaphane. Theranostics.

[18] Gan JC, Ducheyne P, Vresilovic EJ, Swaim W, Shapiro IM (2003). Intervertebral disc tissue engineering I: characterization of the nucleus pulposus. Clin. Orthopaedics Related Res..

[19] Meachim G, Cornah MS (1970). Fine structure of juvenile human nucleus pulposus. J. Anat..

[20] Trout JJ, Buckwalter JA, Moore KC, Landas SK (1982). Ultrastructure of the human intervertebral disc. I. Changes in notochordal cells with age. Tissue Cell.

[21] Madhu V (2020). Hypoxic regulation of mitochondrial metabolism and mitophagy in nucleus pulposus cells is dependent on HIF-1α-BNIP3 axis. J. Bone Mineral Res..

[22] Zhao CQ, Wang LM, Jiang LS, Dai LY (2007). The cell biology of intervertebral disc aging and degeneration. Ageing Res. Rev..

[23] Guehring T, Nerlich A, Kroeber M, Richter W, Omlor GW (2010). Sensitivity of notochordal disc cells to mechanical loading: an experimental animal study. Eur. Spine J..

[24] Guehring T (2009). Notochordal intervertebral disc cells: sensitivity to nutrient deprivation. Arthritis Rheumatism.

[25] Park EY, Park JB (2013). High glucose-induced oxidative stress promotes autophagy through mitochondrial damage in rat notochordal cells. Int. Orthop..

[26] Yurube T (2014). Notochordal cell disappearance and modes of apoptotic cell death in a rat tail static compression-induced disc degeneration model. Arthritis Res. Ther..

[27] Lawson LY, Harfe BD (2017). Developmental mechanisms of intervertebral disc and vertebral column formation. Wiley Interdisciplinary Rev. Dev. Biol.

[28] Weiler C (2010). Immunohistochemical identification of notochordal markers in cells in the aging human lumbar intervertebral disc. Eur. Spine J..

[29] Lv F (2014). In search of nucleus pulposus-specific molecular markers. Rheumatology.

[30] Rodrigues-Pinto R (2016). Spatiotemporal analysis of putative notochordal cell markers reveals CD24 and keratins 8, 18, and 19 as notochord-specific markers during early human intervertebral disc development. J. Orthop. Res..

[31] Merceron C (2014). Loss of HIF-1α in the notochord results in cell death and complete disappearance of the nucleus pulposus. PLoS ONE.

[32] Xu WN (2019). Mitochondrial NDUFA4L2 attenuates the apoptosis of nucleus pulposus cells induced by oxidative stress via the inhibition of mitophagy. Exp. Mol. Med..

[33] Silagi ES (2018). Bicarbonate recycling by HIF-1-dependent carbonic anhydrase isoforms 9 and 12 is critical in maintaining intracellular pH and viability of nucleus pulposus cells. J. Bone Miner. Res..

[34] Hirata H (2014). A rat tail temporary static compression model reproduces different stages of intervertebral disc degeneration with decreased notochordal cell phenotype. J. Orthop. Res..

[35] Won HY, Park JB, Park EY, Riew KD (2009). Effect of hyperglycemia on apoptosis of notochordal cells and intervertebral disc degeneration in diabetic rats. J. Neurosurg. Spine.

[36] Bai, X. D. et al. (*) Coculture with partial digestion notochordal cell-rich nucleus pulposus tissue activates degenerative human nucleus pulposus cells. *Tissue Eng. Part A* **23**, 837–846 (2017). 10.1089/ten.TEA.2016.0428 28145804

[37] de Vries SA, van Doeselaar M, Meij BP, Tryfonidou MA, Ito K (2016). The stimulatory effect of notochordal cell-conditioned medium in a nucleus pulposus explant culture. Tissue Eng. Part A.

[38] de Vries S, Doeselaar MV, Meij B, Tryfonidou M, Ito K (2019). Notochordal cell matrix as a therapeutic agent for intervertebral disc regeneration. Tissue Eng. Part A.

[39] de Vries SA (2015). Conditioned medium derived from notochordal cell-rich nucleus pulposus tissue stimulates matrix production by canine nucleus pulposus cells and bone marrow-derived stromal cells. Tissue Eng. Part A.

[40] Erwin WM, Islam D, Inman RD, Fehlings MG, Tsui FW (2011). Notochordal cells protect nucleus pulposus cells from degradation and apoptosis: implications for the mechanisms of intervertebral disc degeneration. Arthritis Res. Ther..

[41] Mehrkens A (2017). Notochordal cell-derived conditioned medium protects human nucleus pulposus cells from stress-induced apoptosis. Spine J..

[42] Wang J (2018). Polydatin suppresses nucleus pulposus cell senescence, promotes matrix homeostasis and attenuates intervertebral disc degeneration in rats. J. Cell. Mol. Med..

[43] Yi W (2020). HO-1 overexpression alleviates senescence by inducing autophagy via the mitochondrial route in human nucleus pulposus cells. J. Cell. Physiol..

[44] Huang D (2020). Compression-induced senescence of nucleus pulposus cells by promoting mitophagy activation via the PINK1/PARKIN pathway. J. Cell. Mol. Med..

[45] Wang Y (2020). SIRT1 alleviates high-magnitude compression-induced senescence in nucleus pulposus cells via PINK1-dependent mitophagy. Aging.

[46] Li Z (2018). CsA attenuates compression-induced nucleus pulposus mesenchymal stem cells apoptosis via alleviating mitochondrial dysfunction and oxidative stress. Life Sci..

[47] Lin H (2017). Drp1 mediates compression-induced programmed necrosis of rat nucleus pulposus cells by promoting mitochondrial translocation of p53 and nuclear translocation of AIF. Biochem. Biophys. Res. Commun..

[48] Liu J, Yuan C, Pu L, Wang J (2017). Nutrient deprivation induces apoptosis of nucleus pulposus cells via activation of the BNIP3/AIF signalling pathway. Mol. Med. Rep..

[49] Chen S (2018). Critical contribution of RIPK1 mediated mitochondrial dysfunction and oxidative stress to compression-induced rat nucleus pulposus cells necroptosis and apoptosis. Apoptosis.

[50] Xu D (2017). Hydrogen sulfide protects against endoplasmic reticulum stress and mitochondrial injury in nucleus pulposus cells and ameliorates intervertebral disc degeneration. Pharmacol. Res..

[51] Zhao L (2018). Hydrogen peroxide induces programmed necrosis in rat nucleus pulposus cells through the RIP1/RIP3-PARP-AIF pathway. J. Orthop. Res..

[52] Kang L (2020). The mitochondria-targeted anti-oxidant MitoQ protects against intervertebral disc degeneration by ameliorating mitochondrial dysfunction and redox imbalance. Cell Prolif..

[53] Jiang LB (2015). Activation of autophagy via Ca(2+)-dependent AMPK/mTOR pathway in rat notochordal cells is a cellular adaptation under hyperosmotic stress. Cell Cycle.

[54] Liu Y (2019). Aspirin-mediated attenuation of intervertebral disc degeneration by ameliorating reactive oxygen species in vivo and in vitro. Oxid. Med. Cell. Longev..

[55] Feng C (2017). ROS: crucial intermediators in the pathogenesis of intervertebral disc degeneration. Oxid. Med. Cell. Longev..

[56] Smith HL, Mallucci GR (2016). The unfolded protein response: mechanisms and therapy of neurodegeneration. Brain.

[57] Yung HW (2019). Noncanonical mitochondrial unfolded protein response impairs placental oxidative phosphorylation in early-onset preeclampsia. Proc. Natl Acad. Sci. USA.

[58] Naresh NU, Haynes CM (2019). Signaling and regulation of the mitochondrial unfolded protein response. Cold Spring Harbor Perspect. Biol.

[59] Melber A, Haynes CM (2018). UPR(mt) regulation and output: a stress response mediated by mitochondrial-nuclear communication. Cell Res..

[60] Liu Y, Samuel BS, Breen PC, Ruvkun G (2014). Caenorhabditis elegans pathways that surveil and defend mitochondria. Nature.

[61] Kim S, Sieburth D (2018). Sphingosine kinase activates the mitochondrial unfolded protein response and is targeted to mitochondria by stress. Cell Rep..

[62] Pérez, M. J. et al. Loss of function of the mitochondrial peptidase PITRM1 induces proteotoxic stress and Alzheimer’s disease-like pathology in human cerebral organoids. Mol. Psychiatry (2020). 10.1038/s41380-020-0807-4 PMC8758476 32632204

[63] Kenny TC (2017). Selected mitochondrial DNA landscapes activate the SIRT3 axis of the UPR(mt) to promote metastasis. Oncogene.

[64] Seli E, Wang T, Horvath TL (2019). Mitochondrial unfolded protein response: a stress response with implications for fertility and reproductive aging. Fertil. Steril..

[65] Chooi WH, Chan BP (2016). Compression loading-induced stress responses in intervertebral disc cells encapsulated in 3D collagen constructs. Sci. Rep..

[66] Chooi WH, Chan SCW, Gantenbein B, Chan BP (2016). Compression loading induced cellular stress response of intervertebral disc cells in organ culture. Glob. Spine J..

[67] Gogate SS, Fujita N, Skubutyte R, Shapiro IM, Risbud MV (2012). Tonicity enhancer binding protein (TonEBP) and hypoxia-inducible factor (HIF) coordinate heat shock protein 70 (Hsp70) expression in hypoxic nucleus pulposus cells: role of Hsp70 in HIF-1α degradation. J. Bone Miner. Res..

[68] Tsai TT (2007). MEK/ERK signaling controls osmoregulation of nucleus pulposus cells of the intervertebral disc by transactivation of TonEBP/OREBP. J. Bone Miner. Res..

[69] Wang W (2008). Superoxide flashes in single mitochondria. Cell.

[70] Apostolova N, Victor VM (2015). Molecular strategies for targeting antioxidants to mitochondria: therapeutic implications. Antioxid. Redox Signal..

[71] Fukai T, Ushio-Fukai M (2011). Superoxide dismutases: role in redox signaling, vascular function, and diseases. Antioxid. Redox Signal..

[72] Murphy MP (2012). Mitochondrial thiols in antioxidant protection and redox signaling: distinct roles for glutathionylation and other thiol modifications. Antioxid. Redox Signal..

[73] Aldosari S, Awad M, Harrington EO, Sellke FW, Abid MR (2018). Subcellular reactive oxygen species (ROS) in cardiovascular pathophysiology. Antioxidants.

[74] Trachootham D, Lu W, Ogasawara MA, Nilsa RD, Huang P (2008). Redox regulation of cell survival. Antioxid. Redox Signal..

[75] Miyazaki S (2015). Recombinant human SIRT1 protects against nutrient deprivation-induced mitochondrial apoptosis through autophagy induction in human intervertebral disc nucleus pulposus cells. Arthritis Res. Ther..

[76] Li K (2018). Resveratrol protects against sodium nitroprusside induced nucleus pulposus cell apoptosis by scavenging ROS. Int. J. Mol. Med..

[77] Chen J (2019). Protective effects of ginsenoside Rg3 on TNF-α-induced human nucleus pulposus cells through inhibiting NF-κB signaling pathway. Life Sci..

[78] Jin LY (2018). Estradiol alleviates intervertebral disc degeneration through modulating the antioxidant enzymes and inhibiting autophagy in the model of menopause rats. Oxid. Med. Cell. Longev..

[79] Jiao S (2018). Nucleus pulposus cell apoptosis is attenuated by CDMP-2 through regulating oxidative damage under the hyperosmotic environment. Biosci. Rep..

[80] Gu R (2019). Moracin attenuates LPS-induced inflammation in nucleus pulposus cells via Nrf2/HO-1 and NF-?B/TGF-á pathway. Biosci. Rep.

[81] Tang P (2018). Honokiol alleviates the degeneration of intervertebral disc via suppressing the activation of TXNIP-NLRP3 inflammasome signal pathway. Free Radic. Biol. Med..

[82] He R (2018). Melatonin resists oxidative stress-induced apoptosis in nucleus pulposus cells. Life Sci..

[83] Chu H, Yu H, Ren D, Zhu K, Huang H (2016). Plumbagin exerts protective effects in nucleus pulposus cells by attenuating hydrogen peroxide-induced oxidative stress, inflammation and apoptosis through NF-κB and Nrf-2. Int. J. Mol. Med..

[84] Dong Y (2018). Pilose antler peptide attenuates LPS-induced inflammatory reaction. Int. J. Biol. Macromol..

[85] Hoppins S (2014). The regulation of mitochondrial dynamics. Curr. Opin. Cell Biol..

[86] Prieto J (2016). Early ERK1/2 activation promotes DRP1-dependent mitochondrial fission necessary for cell reprogramming. Nat. Commun..

[87] Yao CH (2019). Mitochondrial fusion supports increased oxidative phosphorylation during cell proliferation. eLife.

[88] Li J (2017). Mitochondrial elongation-mediated glucose metabolism reprogramming is essential for tumour cell survival during energy stress. Oncogene.

[89] Youle RJ, van der Bliek AM (2012). Mitochondrial fission, fusion, and stress. Science.

[90] Wai T, Langer T (2016). Mitochondrial dynamics and metabolic regulation. Trends Endocrinol. Metab..

[91] Yang L (2020). Metformin alleviates lead-induced mitochondrial fragmentation via AMPK/Nrf2 activation in SH-SY5Y cells. Redox Biol..

[92] Acin-Perez, R. et al. Ablation of the stress protease OMA1 protects against heart failure in mice. *Sci. Transl. Med*. **10**, (2018). 10.1126/scitranslmed.aan4935 29593106

[93] Amartuvshin O (2020). Aging shifts mitochondrial dynamics toward fission to promote germline stem cell loss. Aging cell.

[94] Gatica D, Lahiri V, Klionsky DJ (2018). Cargo recognition and degradation by selective autophagy. Nat. Cell Biol..

[95] Gustafsson ÅB, Dorn GW (2019). Evolving and expanding the roles of mitophagy as a homeostatic and pathogenic process. Physiol. Rev..

[96] Ashrafi G, Schwarz TL (2013). The pathways of mitophagy for quality control and clearance of mitochondria. Cell Death Differ..

[97] Kirkin V, Rogov VV (2019). A diversity of selective autophagy receptors determines the specificity of the autophagy pathway. Mol. Cell.

[98] Durcan TM, Fon EA (2015). The three ‘P’s of mitophagy: PARKIN, PINK1, and post-translational modifications. Genes Dev..

[99] Rasool S, Trempe JF (2018). New insights into the structure of PINK1 and the mechanism of ubiquitin phosphorylation. Crit. Rev. Biochem. Mol. Biol..

[100] Pickles S, Vigié P, Youle RJ (2018). Mitophagy and quality control mechanisms in mitochondrial maintenance. Curr. Biol..

[101] Zhang Y (2019). Listeria hijacks host mitophagy through a novel mitophagy receptor to evade killing. Nat. Immunol..

[102] Sentelle RD (2012). Ceramide targets autophagosomes to mitochondria and induces lethal mitophagy. Nat. Chem. Biol..

[103] Chu CT (2013). Cardiolipin externalization to the outer mitochondrial membrane acts as an elimination signal for mitophagy in neuronal cells. Nat. Cell Biol..

[104] Zhang Z (2018). Parkin-mediated mitophagy as a potential therapeutic target for intervertebral disc degeneration. Cell Death Dis..

[105] Chen Y (2020). Mfn2 is involved in intervertebral disc degeneration through autophagy modulation. Osteoarthr. Cartil..

[106] Chen Y (2019). Melatonin ameliorates intervertebral disc degeneration via the potential mechanisms of mitophagy induction and apoptosis inhibition. J. Cell. Mol. Med..

[107] Xie L (2019). CircERCC2 ameliorated intervertebral disc degeneration by regulating mitophagy and apoptosis through miR-182-5p/SIRT1 axis. Cell Death Dis..

[108] Wang J (2018). Small molecule natural compound agonist of SIRT3 as a therapeutic target for the treatment of intervertebral disc degeneration. Exp. Mol. Med..

[109] Zhou B (2019). Mitochondrial permeability uncouples elevated autophagy and lifespan extension. Cell.

[110] Knuppertz L, Warnsmann V, Hamann A, Grimm C, Osiewacz HD (2017). Stress-dependent opposing roles for mitophagy in aging of the ascomycete Podospora anserina. Autophagy.

[111] Whitaker RM, Corum D, Beeson CC, Schnellmann RG (2016). Mitochondrial biogenesis as a pharmacological target: a new approach to acute and chronic diseases. Annu. Rev. Pharmacol. Toxicol..

[112] Ploumi C, Daskalaki I, Tavernarakis N (2017). Mitochondrial biogenesis and clearance: a balancing act. FEBS J..

[113] Scarpulla RC, Vega RB, Kelly DP (2012). Transcriptional integration of mitochondrial biogenesis. Trends Endocrinol. Metab..

[114] Wright DC (2007). Exercise-induced mitochondrial biogenesis begins before the increase in muscle PGC-1alpha expression. J. Biol. Chem..

[115] Dorn GW, Vega RB, Kelly DP (2015). Mitochondrial biogenesis and dynamics in the developing and diseased heart. Genes Dev..

[116] Bouchez C, Devin A (2019). Mitochondrial biogenesis and mitochondrial reactive oxygen species (ROS): a complex relationship regulated by the cAMP/PKA signaling pathway. Cells.

[117] Hua W (2020). Icariin protects human nucleus pulposus cells from hydrogen peroxide-induced mitochondria-mediated apoptosis by activating nuclear factor erythroid 2-related factor 2. Biochimica Biophysica Acta Mol. Basis Dis..

[118] Mahrouf-Yorgov M (2017). Mesenchymal stem cells sense mitochondria released from damaged cells as danger signals to activate their rescue properties. Cell Death Differ..

[119] Martin, S. J. Cell biology. Opening the cellular poison cabinet. *Science**330*, 1330–1331 (2010). 10.1126/science.1199461 21127237

[120] Kroemer G, Galluzzi L, Brenner C (2007). Mitochondrial membrane permeabilization in cell death. Physiol. Rev..

[121] Kale J, Osterlund EJ, Andrews DW (2018). BCL-2 family proteins: changing partners in the dance towards death. Cell Death Differ..

[122] Chen D (2016). Metformin protects against apoptosis and senescence in nucleus pulposus cells and ameliorates disc degeneration in vivo. Cell Death Dis..

[123] Lu S (2021). Ferroportin-dependent iron homeostasis protects against oxidative stress-induced nucleus pulposus cell ferroptosis and ameliorates intervertebral disc degeneration in vivo. Oxid. Med. Cell. Longev..

[124] Zhao K (2021). Acid-sensing ion channels regulate nucleus pulposus cell inflammation and pyroptosis via the NLRP3 inflammasome in intervertebral disc degeneration. Cell Prolif..

[125] Cai X (2018). ROS-mediated lysosomal membrane permeabilization is involved in bupivacaine-induced death of rabbit intervertebral disc cells. Redox Biol..

[126] Shirihai OS, Song M, Dorn GW (2015). How mitochondrial dynamism orchestrates mitophagy. Circulation Res..

[127] Palikaras K, Lionaki E, Tavernarakis N (2015). Coordination of mitophagy and mitochondrial biogenesis during ageing in *C. elegans*. Nature.

[128] Palikaras K, Lionaki E, Tavernarakis N (2015). Coupling mitogenesis and mitophagy for longevity. Autophagy.

